# Potential contribution of microbial interaction to stochasticity of microbiota assembly

**DOI:** 10.1093/ismeco/ycag043

**Published:** 2026-02-27

**Authors:** Zhong Yu, Yupeng Liu

**Affiliations:** School of Environmental Science and Engineering, Sun Yat-sen University, Guangzhou 510275, PR China; Guangdong Provincial Key Laboratory of Environmental Pollution Control and Remediation Technology (Sun Yat-sen University), Guangzhou 510275, PR China; Department of Cardiology, Guangdong Provincial People's Hospital (Guangdong Academy of Medical Sciences), Southern Medical University, Guangzhou 510080, China; Guangdong Cardiovascular Institute, Guangdong Provincial People's Hospital, Guangdong Academy of Medical Sciences, Guangzhou 510080, China

**Keywords:** varying interaction, community assembly, stochastic process, relative importance, iCAMP

## Abstract

Microbial interaction has been widely acknowledged as the deterministic factor for microbiota assembly. However, in the microbial environment, such interactions are highly variable and may lead to the random birth, death, and reproduction of neighboring microbes, potentially contributing to stochastic assembly processes. We have developed a Go-board model to simulate and investigate how microbiota assembly is affected by different types of interacting species, interaction strength, and community structure. The infer Community Assembly Mechanisms by Phylogenetic-bin-based null model analysis (iCAMP) shows that the strong inhibitory effect of deterministic species on stochastic species can lead to a switch of the dominant assembly process from heterogeneous selection to drift. On the other hand, the effects of the inhibition of stochastic species on their deterministic neighbors enhances the relative importance of dispersal limitation in communities with small deterministic populations. The mutual inhibition between deterministic and stochastic species further enhances the stochasticity of microbiota assembly, but this effect is canceled out by the effects of interspecific facilitation and deterministic species with large phylogenetic distance. Our study sheds novel light on the role of microbial interaction in stochastic assembly processes, which is contradictory to the traditional niche-based theory. These findings highlight the importance of assessing interspecific interaction for predicting the true roles of different assembly processes in future studies.

## Introduction

Microbes commonly live in communities and assemble high-diversity microbiota with multiple functions. Such complex microbiota play significant roles in human health, environmental integrity, and engineering system performance [1]. Understanding the community assembly mechanism is a long-standing goal for microbial ecology [2, 3] and is also the key for designing and constructing efficient and stable microbiota for specific engineering purposes [4].

It has been widely acknowledged that during microbiota assembly, the microbe community will experience deterministic selection and three stochastic processes, including drift, diversification, and dispersal [5, 6]. Specifically, selection arises from abiotic and biotic effects, including environmental conditions, species traits, and interspecific interactions, which are considered deterministic [3]. Previous studies suggest that such determinism indicates that there is no random variation in demographic rates of community members under the selection pressures [7, 8], which results in the predictable outcomes of community assembly. Take the prediction of abundances of obligate anaerobes like methanogen in anaerobic and aerobic environments as an example. If dissolved oxygen does serve as a deterministic factor for methanogen, the demographic rate of methanogen in an anaerobic environment should be predictably higher than that in an aerobic environment. Therefore, without the need for any high-throughput sequencing one can expect that the relative abundance of methanogen in an aerobic environment is lower than that in an anaerobic environment.

**Figure 1:**
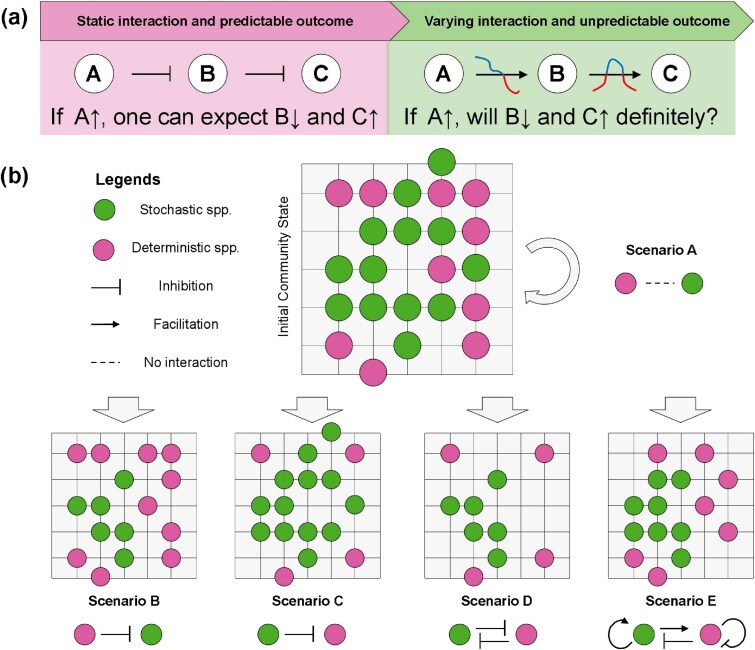
The potential contribution of varying and short-range interaction to the stochastic microbiota assembly. (a) The hypothesis of current study: the varying and short-range interaction may not always result in the deterministic community pattern as predicted by interaction graph. (b) The schematic illustration of microbial assembly with different interspecific interactions, including scenario A (no interspecific interaction), B (inhibition of deterministic species on stochastic species), C (inhibition of stochastic species on deterministic ones), D (mutual inhibition between deterministic and stochastic species), and E (inhibition and facilitation among deterministic and stochastic species).

With respect to interspecific interaction, niche-based theory suggests that this process also serves as a deterministic factor for microbiota assembly, with the inhibition chain as an example (Fig. 1a). If microbial interaction remains stable, one can expect that the demographic rate of downstream species will be higher when the upstream inhibitor is decreased, but lower otherwise. Hence, upon the blooming of its competitors, the decline in the relative abundance of a species can be predicted from a graph-based perspective. However, recent studies suggest that such interspecific interactions can be highly variable even under the same environmental conditions [9, 10]. For example, a previous study found that *Ketogulonicigenium vulgare* and *Bacillus megaterium*can facilitate the growth of each other by the exchange of metabolites [11]. However, upon increased abundance of *B. megaterium, K. vulgare* will experience nutrient exhaustion and thus its facilitative effect on *B. megaterium* will be switched to an inhibitory one. As such, *B. megaterium* can exhibit varying demographic rates. Moreover, microbes in a structured environment (e.g. biofilm) or dense community interact only within a short range, i.e, a few cell lengths [12]. Given the ubiquitous heterogeneity of micro-environments in microbiota [13], microbial individuals may impose different effects on the proximal neighbors at local sites but have limited effects on the distant cells. As such, the cells at different locations could exhibit different growth and dispersal rates even when they share the same taxonomy. That is, a local cell is less likely to grow when encountering more inhibitors. We hypothesize that this process should drive the local microbe community into distinct structures [9], and under these conditions the microbiota assembly may be stochastic at the local level. Therefore, it is important to evaluate the effects of varying and short-range interspecific interactions on microbiota assembly, which are key to predicting the true relative importance of different ecological processes.

Inspired by the Chinese chess game of Go, a Go-board model was developed to simulate microbiota assembly under varying interspecific interaction. Like many ecological models (e.g. Sloan’s Neutral Community Model), our Go-board model is a simplified model that simulates the snapshot of microbiota dynamics with only interspecific interaction in the ideal environment. Based on this information, we used the infer Community Assembly Mechanisms by Phylogenetic-bin-based null model analysis (iCAMP) to compare the relative importance of different assembly processes. The main objectives of this study were to answer the following questions: (i) Does the microbial interaction variability have stochastic effect on microbiota assembly? (ii) Is the stochasticity of microbial interaction species dependent? (iii) Does the relative importance of stochasticity of interspecific interaction change in different microbiota? Our results suggest that interspecific interaction plays an important role in the stochastic processes during microbiota assembly in the ideal environment simulated by the Go-board model. The specific effects of interspecific inhibition and facilitation on microbiota assembly depend on the types of source and target species in the interaction, as well as the initial structure of the local community. These ideal observations challenge the applicability of niche-based assumptions regarding the deterministic role of interspecific interaction in the practical microbiota assembly.

## Materials and methods

### Simulation of interspecific interaction during microbiota assembly

A total of 5 scenarios were proposed to represent the effects of interspecific interaction on the outcome of microbiota assembly. Here, interspecific interaction refers to inhibition and facilitation effects on a target species by a source species, which can remove and retain the target species encountering the source species, respectively. To simulate the outcome of microbiota assembly, we developed a Go-board model to mimic the short-range interspecific interaction in the microbial community (Fig. 1b). Each microbial individual was treated as a piece and randomly placed on a position of the board *p_i,j_*, where it would only interact with the adjacent individuals. Notably, *p_i,j_* equals D (or S) when the position is occupied by a deterministic (or stochastic) species, but equals 0 when the piece is captured. We defined the short-range interaction as the effect of a species at *p_i,j_* on the species within a radius of *r*, where *r* is a finite and positive integer and was set at 1 in this study for simplicity. With larger value of *r*, one can simulate the more distant interaction between microbial cells caused by passive volatiles diffusion.

#### Scenario A: microbial assembly without interspecific inhibition

During the microbiota assembly of a local community, some species are controlled by the abiotic filtering [3], and these deterministic species constitute the native community in a given environment. In comparison, some species depend on immigration from the global metacommunity and their assemblies are completely stochastic. While these native and immigrant species are expected to compete for the shared resource in the local environment, they could coexist in a spatially “frozen” pattern in a structured environment [14]. In this scenario, the expected structure of the local community would depend on the initial abundances of the native species in the local community and immigrant species from the global metacommunity (i.e., *p’_i,j_* = *p_i,j_*).

#### Scenario B: microbial assembly with inhibition of deterministic species on stochastic species

In some cases, immigrant species can occupy a habitat, i.e. *p_i,j_* = S. However, these species may not be well adapted to the local environment and may thus display low metabolic activity [15]. As a result, these specis should have limited effects on the native species that survive the local deterministic selection. Instead, their colonization and growth may be inhibited by native microbiota, e.g. the resistance of human gut microbiota to invading pathogens [16]. The outcome of this scenario, *p’_i,j_*, is that stochastic species in close proximity to deterministic species will be eliminated, so we can write equation (1):


1 \begin{eqnarray*} {p}^{{\prime}}i,j=\left\{\begin{array}{@{}cc}0& \mathrm{if}\ \exists p\in \left\{{p}_{i+1,j},{p}_{i-1,j},{p}_{i,j+1},{p}_{i,j-1}\right\}\ \mathrm{and}\ p={\mathrm{D}}^{\prime }\ \\{}\mathrm{S}& \mathrm{otherwise}\ \end{array}\right. \end{eqnarray*}

where {*p_i + 1,j_, p_i-1,j_, p_i,j + 1_, p_i,j-1_*} represents a non-zero set of positions adjacent to *p_i,j_* and D′ is the deterministic species (D0, D1, or both) that can inhibit the given stochastic species. It can be expected that if more stochastic species are inhibited by D′, the *p’_i,j_* is more inclined to be zero.

#### Scenario C: microbial assembly with inhibition of stochastic species on deterministic species

In some cases (*p_i,j_* = D), some immigrant microbes (e.g. phages) can directly kill the sensitive species in the native community [17]. Moreover, some invaders may compete for the shared resources and thus lead to the extinction of native species in local communities [18]. As such, it is expected that the outcome of the scenario *p’_i,j_* is the opposite of scenario B, in which the deterministic species are eliminated during their encounters with stochastic species


2 \begin{eqnarray*} {p}^{{\prime}}i,j=\left\{\begin{array}{@{}cc}0& \mathrm{if}\, \exists p\in \left\{{p}_{i+1,j},{p}_{i-1,j},{p}_{i,j+1},{p}_{i,j-1}\right\}\ \mathrm{and}\ p={\mathrm{S}}^{\prime}\kern0.5em \\{}\mathrm{D}& \mathrm{otherwise}\ \end{array}\right. \end{eqnarray*}

where {*p_i + 1,j_, p_i-1,j_, p_i,j + 1_, p_i,j-1_*} represents a non-zero set of positions adjacent to *p_i,j_* and S′ represents the stochastic species that inhibit the deterministic species (D0, D1, or both). If the stochastic species can inhibit more deterministic species, the *p’_i,j_* is more inclined to be zero.

#### Scenario D: microbial assembly with mutual inhibition between deterministic and stochastic species

In other cases, both native and immigrant species could inhibit each other by chemical warfare [19]. However, it should be noted that some species may exhibit antimicrobial resistance, e.g. antibiotic resistance genes [20] or antimicrobial peptides resistance genes [21]. The outcome of the scenario *p’_i,j_* is that the local community members will be eliminated if they are sensitive to antimicrobial substances secreted by their neighbors


3 \begin{eqnarray*} {p}^{{\prime}}i,j=\left\{\begin{array}{@{}cc}0& \mathrm{if}\, \exists p\in \left\{{p}_{i+1,j},{p}_{i-1,j},{p}_{i,j+1},{p}_{i,j-1}\right\}\ \mathrm{and}\ p=\mathrm{I}\\{}{p}_{i,j}& \mathrm{otherwise}\end{array}\right. \end{eqnarray*}

where {*p_i + 1,j_, p_i-1,j_, p_i,j + 1_, p_i,j-1_*} represents a non-zero set of positions adjacent to *p_i,j_. I* is the given inhibitor of the species in position *p_i,j_*.

#### Scenario E: microbial assembly with inhibition and facilitation among species

In addition to interspecific inhibition, cooperation is common among different microbes [22]. Such positive interaction can help local microbes to form a close cooperative loop and use the limited resources more efficiently [23, 24]. However, microbial interaction can switch from facilitation to inhibition and vice versa, according to the environmental resource and toxicity [25]. Therefore, the outcome of this scenario should depend on the strengths of both inhibition and facilitation among different microbes. More specifically, a microbe at position *p’_i,j_* is expected to be eliminated if it is surrounded by more inhibitors than cooperators but can defend its niche against inhibitors if adjacent to more cooperators


4 \begin{eqnarray*} {p}^{{\prime}}i,j=\left\{\begin{array}{@{}cc}0& \mathrm{if}\ {\mathrm{N}}_{\mathrm{C}}<{\mathrm{N}}_{\mathrm{I}}\\{}{p}_{i,j}& \mathrm{otherwise}\end{array},\right. \end{eqnarray*}

where *N_C_* and *N_I_* represent the number of co-operators and inhibitors belonging to the non-zero set $\left\{{p}_{i+1,j},{p}_{i-1,j},{p}_{i,j+1},{p}_{i,j-1}\right\}$.

#### The microbiota assembly simulation with the Go-board model

After placing the deterministic and stochastic species on the Go-board, we randomly chose the interacting microbes from the local community. For example, for the simulation of Scenario B, different proportions (i.e. $\frac{\mathrm{number}\ \mathrm{of}\ \mathrm{selected}\ \mathrm{species}}{\mathrm{total}\ \mathrm{number}\ \mathrm{of}\ \mathrm{stochastic}\ \mathrm{species}}\times 100\%$) ranging from 10% to 90%, with 20% intervals of the stochastic species, were chosen to be inhibited by the deterministic species. The higher proportion of inhibited stochastic species indicated the stronger inhibition in the local community. Under the given proportion of interacting species, the same set of inhibitors or cooperators was used for different communities to estimate their effects on microbiota assembly. Next, we performed iteration over the whole Go-board and captured the pieces according to the rules from 2.1.1 to 2.1.5. Finally, we summarized the taxonomy and abundance of the remaining pieces. Notably, the Go-board model forgoes the assumptions of dispersal and growth of the interacting microbes in the local community that have been widely made by the spatial ecology models [26]. Accordingly, we simulated only a single time step so that we could rule out the effect of local dispersal and growth on the outcome of the Go-board model.

### Simulated model

**Figure 2:**
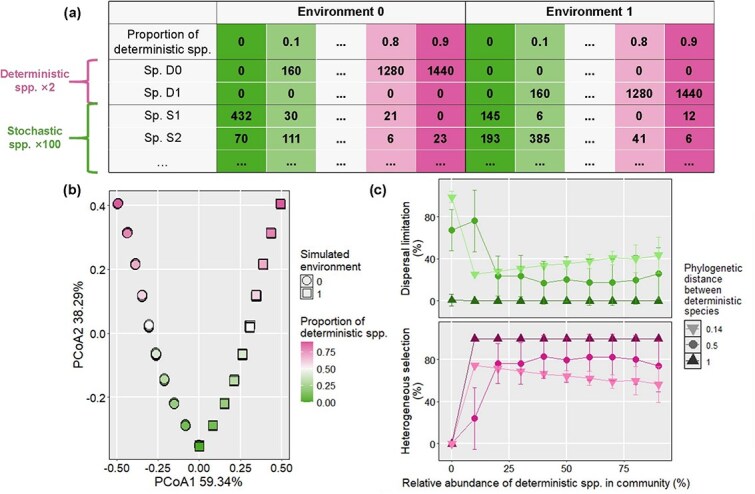
The assembly processes in the simulated microbiota. (a) The simulated metacommunity following the mZSM distribution. (b) The PCoA result of simulated microbiota under different environments. The shape and color of each dot represent the simulated environment and proportion of deterministic species of a local community, respectively. (c–d) The relative importance of heterogeneous selection and dispersal limiation for the assembly of simulated community for *d_det, det_* = 0.5. The error bar represents the standard deviation of relative importance for a given ecological process.

A total of 2000 simulated communities with different proportions of deterministic species were generated in the current study (Fig. 2a). Only 2 types of deterministic species (D0 and D1) and environments (Environment 0 and 1) were simulated and there were 1000 simulations for the 2 environments, respectively. Therein, D0 would be present only in the simulated community in environment 0, while D1 would be enriched only in environment 1. The relative abundances of deterministic species were designed as 0%, 10%, 30%, 50%, 70%, and 90%. We expected that higher relative abundance of deterministic species in the simulated community would lead to higher relative importance of deterministic process in microbiota assembly. To further simulate the stochastic species, we first used Hubbell’s Unified Neutral Theory Model to generate a metacommunity following the metacommunity zero-sum multinomial (mZSM) distribution as previously described [3]. More specifically, a metacommunity containing 10 000 species was generated by the R package “sads,” with total abundance *J* of 10^8^ and biodiversity index *θ* for mZSM of 5000 as suggested by Ning et al. [3]. Accordingly, we simulated the stochastic species for all local communities by randomly drawing 100 species from the metacommunity. The Sloan’s Neutral Model was used to determine the occurrence frequencies of these stochastic species based on their average relative abundances across 2000 simulated communities. The total abundance of a simulated community was set as 1600 to cover the 40 × 40 Go-board so that each species could have at least 1 individual on the Go-board under all circumstances.

Moreover, we built a phylogenetic tree based on the assumption that phylogenetic distance approximated niche difference of simulated species across short phylogenetic distances (< 0.14) [5]. Therefore, the phylogenetic distance *d_det, det_* between deterministic species with large niche difference could be a random number between 0.14 and 1. In the current study, we have investigated the effect of different phylogenetic distances between deterministic species (*d_det, det_* = 0.14, 0.5, and 1) on microbiota assembly processes. Stochastic species could survive in both simulated environments and displayed zero habitat difference. Accordingly, these species should be close relatives, whose maximum phylogenetic distance max(*d_sto, sto_*) was lower than 0.14. Moreover, the minimum phylogenetic distance between deterministic and stochastic species was set to be (*d_det, det_* − max(*d_sto, sto_*))/2.

### The iCAMP analysis

The relative importance of ecological processes for the simulated microbiota was analyzed using the iCAMP package of R [27]. Specifically, the community members were divided into different bins based on the simulated phylogenetic distance. Given the maximum *d_sto, sto_* of 0.14 in a simulated community, the phylogenetic signal threshold was set to be 0.1 so that the stochastic species could be divided into different bins. Such threshold establishment should help us to investigate the differentiation of assembly processes of stochastic species after the interspecific interaction. Accordingly, pairwise comparison was conducted between every 2 bins to calculate the beta Net Relatedness Index (βNRI) and modified Raup-Crick (RC) metric. More specifically, the βNRI is calculated based on the observed and null beta mean pairwise distances βMPDs, which are calculated by taxon abundances and phylogenetic distance [28] according to this equation:


5 \begin{eqnarray*} \mathrm{\beta} \mathrm{MPD}=\frac{\sum_{\mathrm{i}}^{{\mathrm{S}}_{\mathrm{k}}}{\sum}_{\mathrm{j}}^{{\mathrm{S}}_{\mathrm{k}}}{\mathrm{f}}_{\mathrm{i}\mathrm{u}}{\mathrm{f}}_{\mathrm{j}\mathrm{v}}{\mathrm{d}}_{\mathrm{i}\mathrm{j}}}{\sum_{\mathrm{i}}^{{\mathrm{S}}_{\mathrm{k}}}{\sum}_{\mathrm{j}}^{{\mathrm{S}}_{\mathrm{k}}}{\mathrm{f}}_{\mathrm{i}\mathrm{u}}{\mathrm{f}}_{\mathrm{j}\mathrm{v}}} \end{eqnarray*}

where taxa *i* and *j* are the elements of *k^th^* bin. The *f_iu_* and *f_jv_* are the observed abundances of taxa *i* and *j* in communities *u* and *v*, respectively. The *d_ij_* is the phylogenetic distance between *i* and *j*. The null βMPDs are calculated by shuffling the *f_iu_* and *f_jv_* of different taxa. The difference between 2 communities is regarded to be governed by deterministic process if the observed βMPD lies outside the 95% reference interval (RI) of the null βMPDs. On the other hand, the RC metric represents the probability that observed number of taxa common to 2 communities will be greater than the expected numbers based on the null hypothesis [29]. The RC metric is further reduced by 0.5 and multiplied by 2 to be standardized to range from −1 to 1 [30]. The resulting βNRI and modified RC metric were further used to calculate the operator that counted whether an ecological process dominated a given bin. In brief, the thresholds at βNRI of ±1.96 and RC of ±0.95 were applied for identifying the governing process in a bin, including heterogeneous selection (βNRI >1.96), homogeneous selection (βNRI < −1.96), dispersal limitation (|βNRI| ≤ 1.96 and RC > 0.95), homogenizing dispersal (|βNRI| ≤ 1.96 and |RC| ≤ 0.95), and drift (|βNRI| ≤ 1.96 and RC < −0.95). Finally, the relative importance of ecological process in the turnover between 2 local communities was calculated by the sum of the aforementioned operators of all bins. We have developed an R script to summarize the relative importance of the *τ*th ecological process *RI_τuvDI_* in the turnover between the 2 communities *u* and *v* as a function of the relative abundance of deterministic species *D* and the proportion of inhibited species *I* in a community. Notably, the communities *u* and *v* were from different environments so that we can estimate the true relative importance of heterogeneous selection. The relative importance of each ecological process was shown as the mean ± standard deviation unless otherwise indicated. The *t*-test was used to estimate the effect of interspecific interaction on *RI_τuvDI_* under the given *D* and *I*. The interspecific interaction is regarded as significant to affect microbiota assembly if the *P*-value is less than .05.

## Results

### The relative importance of assembly processes without interspecific interaction

We first investigated the assembly of simulated microbiota without interspecific interaction (Fig. 2). As shown in Fig. 2b, the simulated communities were found to separate significantly as a function of the relative abundance of deterministic species. Such result indicates that with increasing deterministic species, the heterogeneity of local community should increase under different simulated environments. As expected, the pairwise comparison of local community structure by iCAMP showed that dispersal limitation dominated the assembly of simulated community without deterministic species, with an average relative importance of 98.74 ± 5.31% and 67.26 ± 19.70% at *d_det, det_* of 0.14 and 0.5, respectively (Fig. 2c). However, when *d_det, det_* equaled 1, the relative importance of dispersal limitation became negligible (0.59 ± 5.77%) and homogeneous selection (99.40 ± 0.00%) became the dominant process for microbiota assembly. We reasoned that the large *d_det, det_* would result in the null βMPD value being larger than the observed βMPD without the deterministic species. That is, the observed dissimilarity between the microbiota without deterministic species is much smaller than the null expected dissimilarity [3]. Such dissimilarity will be identified to be governed by the homogeneous selection by iCAMP, thus leading to an accuracy of iCAMP inference ranging from 0.93 to 0.99 [27]. Moreover, the effects of heterogeneous selection (0.00 ± 0.00%) and drift (ranging from 0.01 ± 0.00% to 1.26 ± 0.00%; Fig. S1) on community assembly were negligible.

The relative importance of dispersal limitation exhibited a dramatic decline from 98.74 ± 5.31% to 25.38 ± 0.71% at *d_det, det_* of 0.14 when the relative abundance of deterministic species increased from 0% to only 10%. With increasing relative abundance of deterministic species, the relative importance of dispersal limitation was found to increase to 43.51% ± 17.23% when deterministic species accounted for 90% of the population. This finding could be attributed to the fact that at a small *d_det, det_* of 0.14, there may not be large difference between observed and null expected dissimilarities for the microbiota with high abundance of deterministic species. Notably, such effects could be eliminated if the deterministic species were phylogenetically distant (*d_det, det_ =* 0.5 or 1). For example, similar results had been obtained for the dispersal limitation at *d_det, det_* of 0.5, but its relative importance remained lower than 30%. Moreover, the relative importance of dispersal limitation remained at 0.00 ± 0.00% when *d_det, det_* = 1. On the other hand, heterogeneous selection became the dominant assembly process as expected, but its average relative importance also remained comparatively stable when deterministic species accounted for more than 10% of the total population. Moreover, the relative importance of homogeneous selection, homogenizing dispersal, and drift was 0.00% ± 0.00% in the presence of deterministic species in a simulated community (Fig. S1). Overall, the relative importance of different ecological processes of microbiota assembly without interspecific interaction can serve as the ground truth for other scenarios. If interspecific interaction serves as the deterministic process according to niche-based theory, we can expect increased relative importance for deterministic selection and decreased importance for stochastic processes in other scenarios compared to those in scenario A. In the remainder of the study, we compared the relative importance of heterogeneous selection and dispersal limitation for microbiota assembly with different interactions at *d_det, det_* of 0.5.

### Assembly of simulated microbiota with inhibition of deterministic species on stochastic species

**Figure 3:**
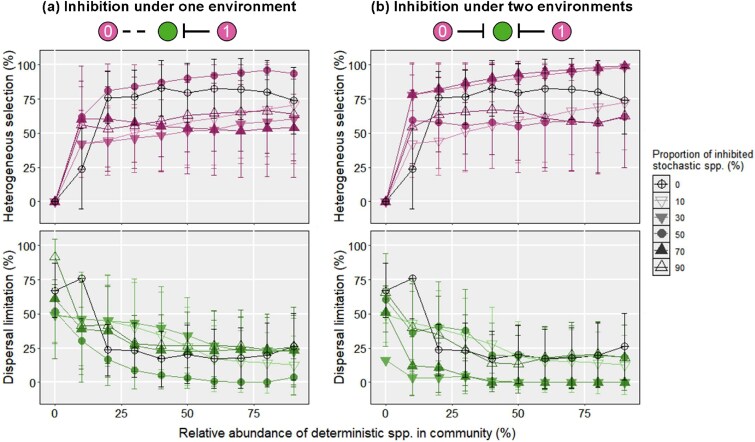
Assembly processes under inhibition of deterministic species on stochastic species for *d_det, det_* = 0.5. (a–b) The relative importance of heterogeneous selection and dispersal limitation as the functions of the proportions of deterministic species and inhibited stochastic species under one (a) and both (b) simulated environments. The black line represents the relative importance of a given ecological process during the microbiota assembly without interaction. The error bar represents the standard deviation of relative importance for a given ecological process.

Fig. 3a shows the varying relative importance of ecological processes when only 1 deterministic species inhibited the stochastic species (i.e. scenario B), while there was no interaction between the other deterministic species and its stochastic neighbors. Compared to scenario A, in scensrio B we observed a similar decline in the relative importance of dispersal limitation and increased dominance of heterogeneous selection with increasing abundances of deterministic species in local communities. However, the average relative importance of heterogeneous selection was 1.31-fold higher than those in scenario A only when 50% of the stochastic species were inhibited (*P* value <.05). More surprisingly, heterogeneous selection was found to hold a less important role (0.74 ~ 0.90-fold; *P* value <.05) for microbiota assembly when 10%, 30%, 70%, and 90% of the stochastic species were inhibited and deterministic species accounted for more than 20% of population. Similarly, when both deterministic species inhibited their stochastic neighbors, the average relative importance of heterogeneous selection was not always higher than that in scenario A (0.90 ~ 1.37-fold; Fig. 3b). This result could be attributed to the random choice of inhibited stochastic species. That is, some inhibited stochastic species may be high in abundance and thus their extinctions contribute more to the variation in the assembly process of the whole local community.

The inhibition of stochastic species did not always decrease the relative importance of dispersal limitation compared to that in scenario A. Moreover, drift became more important for microbiota assembly when deterministic species accounted for more than 30% of population (Fig. S2). We reasoned that under the high encounter rate between stochastic species and their deterministic inhibitor, the diversity of local community would decrease dramatically (Fig. S3). This decrease should result in the sparse matrices of the community profile and short beta mean pairwise distance between the 2 communities, which should further lead to |βNRI| between the 2 communities below 1.96. Overall, the result of scenario B suggests that the high encounter rate between stochastic species and their deterministic inhibitor could reduce the relative importance of heterogeneous selection due to the loss of biodiversity.

### The contribution of inhibition by stochastic species to microbiota assembly

**Figure 4:**
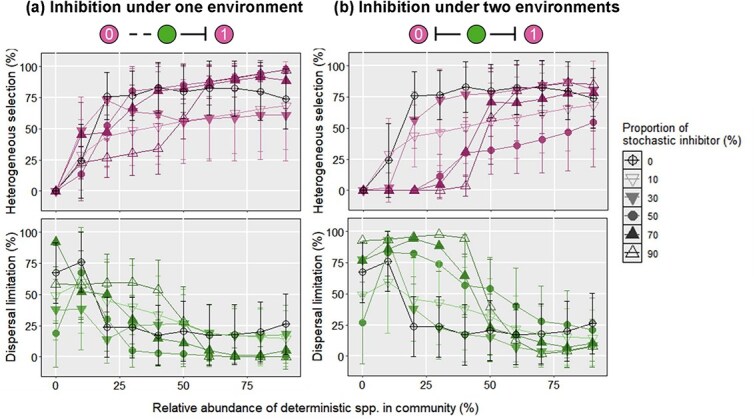
Assembly processes under inhibition of deterministic species on stochastic species for *d_det, det_* = 0.5. (a–b) The relative importance of heterogeneous selection and dispersal limitation as the functions of the proportions of deterministic species and stochastic inhibitors under one (a) and both (b) simulated environments. The black line represents the relative importance of a given ecological process during the microbiota assembly without interaction. The error bar represents the standard deviation of relative importance for a given ecological process.

Figure 4 displays the relative importance of dispersal limitation and heterogeneous selection during microbiota assembly under inhibition on deterministic species by their stochastic neighbors (i.e. scenario C). Interestingly, with a high proportion of stochastic inhibitors in the simulated community (90%), dispersal limitation retained a high relative importance for the assembly of microbiota with small deterministic population (20% ~ 40%). Consistent with this result, heterogeneous selection played the less important role for microbiota assembly. Notably, dispersal limitation was observed with a higher dominance in microbiota assembly if the stochastic species could inhibit both deterministic species (Fig. 4b). Moreover, the relative importance of heterogeneous selection was lower during the assembly of these microbiota, especially when the relative abundance of deterministic species was lower than 40%. We reasoned that the inhibition of deterministic species could result in the reduction of the population of phylogenetically distant species, rather than the loss of overall diversity in local communities in scenario B. Therefore,this inhibition may reduce the observed βMPDs for all turnovers between the 2 local communities. In both cases, such effects of stochastic inhibition on microbiota assembly were canceled out when deterministic species dominated the total population (>50%). Together, these results show that inhibition of deterministic species can switch the assembly process from deterministic to stochastic under certain circumstances.

### The effect of mutual inhibition between deterministic and stochastic species on microbiota assembly

**Figure 5:**
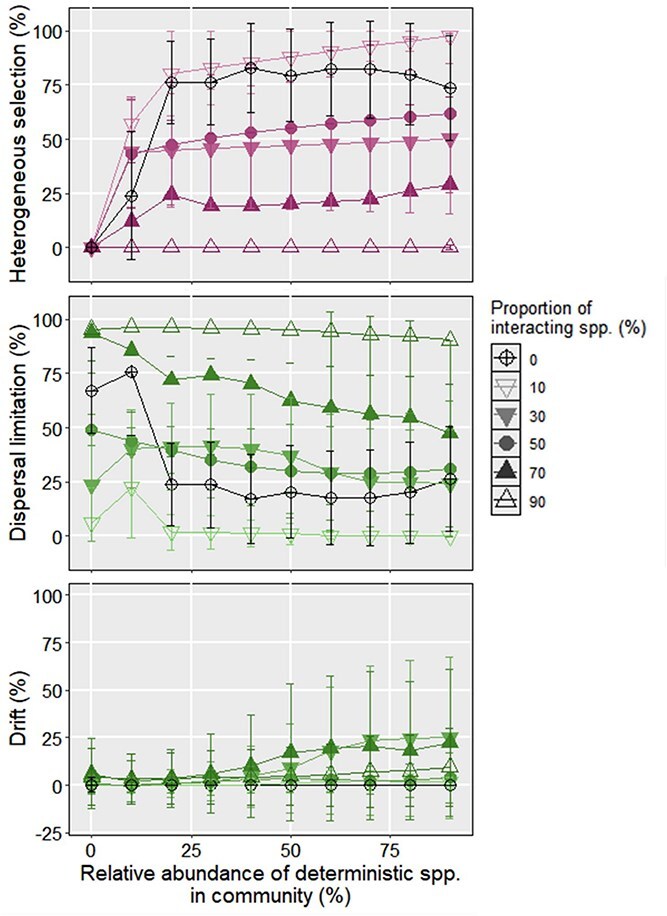
The relative importance of heterogeneous selection, dispersal limitation, and drift during microbiota assembly under mutual inhibition between deterministic species and stochastic species for *d_det, det_* = 0.5. The black line represents the relative importance of a given ecological process during the microbiota assembly without interaction. The error bar represents the standard deviation of relative importance for a given ecological process.

The different contributions of inhibition by deterministic and stochastic species to ecological stochasticity raised an intriguing question about their joint effect on microbiota assembly (Fig. 5). When only 10% of total microbiota were inhibited by deterministic and stochastic species, the relative importance of dispersal limitation was reduced and heterogeneous selection became more important for microbiota assembly as predicted by the traditional niche-based theory. However, with more inhibited species, we observed significantly higher and lower relative importance of dispersal limitation and heterogeneous selection, respectively, compared to the species in scenario A for most cases. Moreover, the mutual inhibition on deterministic and stochastic species may lead to a community dissimilarity as expected levels of phylogenetic turnover [5], thus increasing the relative importance of drift when deterministic species dominated the community (>50%). These observations suggest that the strong inhibition by deterministic and stochastic species can jointly enhance the stochasticity of microbiota assembly rather than cancel each other out. However, it should be noted that the variation of assembly processes followed different patterns in some cases. For example, when interacting species account for 50% of total species, the relative importance of heterogeneous selection was higher than that for 30% of the species. This finding was possibly owing to the random choice of inhibited species during simulation of microbiota assembly.

### Effect of varying interspecific cooperation on microbiota assembly

**Figure 6:**
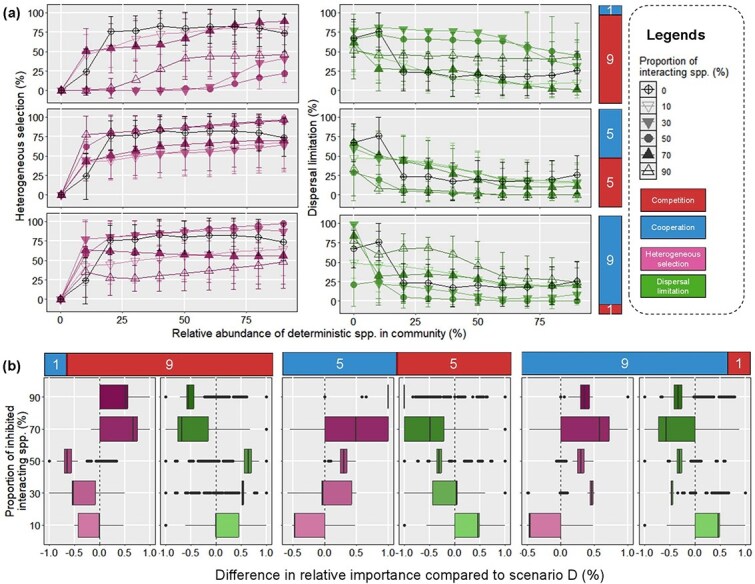
The relative importance of ecological processes during microbiota assembly with different ratios of interspecific inhibition and facilitation for *d_det, det_* = 0.5. (a) The black line represents the relative importance of a given ecological process during the microbiota assembly without interaction. The error bar represents the standard deviation of relative importance for a given ecological process. (b) The difference in the relative importance of heterogeneous (purple) and dispersal limitation (green) between scenario D and E.

While we observed a strong effect of interspecific inhibition between deterministic and stochastic species on microbiota assembly, we hypothesized that their cooperation should cancel out such effect. Figure 6a shows the relative importance of ecological processes under different ratios of inhibition and facilitation on given proportions of community members. When inhibition dominated the simulated community (90%), we found that dispersal limitation held a less important role when interacting species accounted for ≥70% of total species than it did in scenario D (Fig. 6b). Moreover, there was significant enhancement in the relative importance of heterogeneous selection in the microbiota assembly under these circumstances. These results suggest that facilitation can switch the stochastic assembly process to the deterministic one as suggested by the traditional niche-based theory. However, we observed an opposite trend when there were limited interacting species (≤50%). Analogous results had been observed when facilitation became equally dominant with inhibition (50%) or dominated the community (90%). However, compared to scenario D, lower and higher importance of dispersal limitation and heterogeneous selection were observed only when interacting species accounted for 10% of total species, respectively. This finding further indicates that strong facilitation can cancel out the influence of inhibition on microbiota assembly, possibly depending on biodiversity variation induced by the complex interaction.

### Effect of phylogenetic distance on the iCAMP analysis with the Go-board model

**Figure 7:**
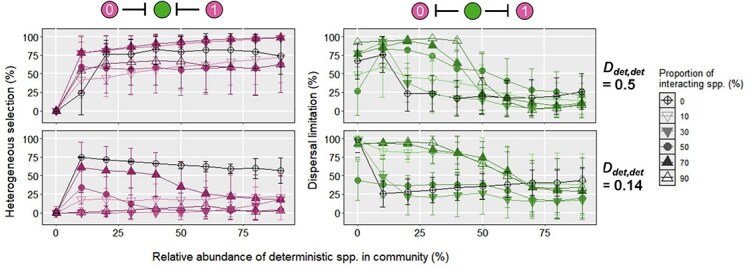
The comparisons between the relative importance of heterogeneous selection and dispersal limitation at *d_det, det_* = 0.14 and 0.5. The black line represents the relative importance of a given ecological process during the microbiota assembly without interaction. The error bar represents the standard deviation of relative importance for a given ecological process.

To further test the generalization of the aforementioned finding, we compared the relative importance of some ecological processes at *d_det, det_* of 0.14 and 0.5, respectively (Fig. 7). We did not consider the results at *d_det, det_* of 1 so that we could make a fair comparison, because the relative importance of heterogeneous selection was 100% for most cases. Our results showed that with the smaller phylogenetic distance between deterministic species (*d_det, det_* = 0.14), the average relative importance of heterogeneous selection was lower than that at *d_det, det_* of 0.5. Interestingly, when the deterministic species accounted for more than 60% of the total population, the inhibition of deterministic species on stochastic species led to a relative importance of heterogeneous selection lower than 25%. We reasoned that such phenomena were attributable to the finding that the small phylogenetic distance between deterministic and stochastic species led to the small observed βMPD. As such, it was more likely that the observed βMPD lay within the 95% RI of the null βMPDs, and the stochastic process was identified as the dominated process. Compared to the results at *d_det, det_* of 0.5, analogous results had been obtained at *d_det, det_* of 0.14 for scenario C when both deterministic species were inhibited by the stochastic neighbors. More specifically, when the deterministic species accounted for 50% of the total population, the average relative importance of dispersal limitation (*d_det, det_* = 0.14) was higher than 50% if 10%, 70%, and 90% of the stochastic species were inhibited by deterministic species. In comparison, the average relative importances of dispersal limitation at *d_det, det_* of 0.5 were lower than 50% for most cases. Overall, these results imply that the larger phylogenetic distance between deterministic species can lead to the lower relative importance of stochastic processes.

## Discussion

Microbes commonly assemble into complex communities by the joint force of deterministic selection and stochastic processes [5, 6]. It is widely acknowledged that microbial interaction serves as a deterministic factor [3, 31], which should lead to limited randomness in demographic rates of community members [7]. For example, in an inhibition chain (Fig. 1a), one may expect that inhibition of midstream species by the upstream species can lead to the blooming of downstream species. However, the interspecific interactions in communities are highly variable with regard to cellular traits, cell density, community structure, and surrounding environment [10]. More importantly, because microbes can only affect their neighbors in a short range i.e. a few cell lengths [12], the sensitive species may survive the killing by its inhibitor if there is no encounter. Consequently, even the static interaction may not always generate the deterministic local community pattern as expected. This finding raises an intriguing question as to whether such variation of interaction outcome at local scale could contribute to the stochasticity of microbiota assembly. To address this concern, we have developed a Go-board model to investigate the effect of different interspecific interactions on simulated microbiota assembly.

Unlike most models in spatial ecology [26, 32], the Go-board model forgoes the assumption that microbes can grow and disperse across the community. The microbes are treated as the static pieces on the Go-board and can only interact with their neighbors. This confers an advantage to the Go-board model that enables us rule out the influence of microbial growth and dispersal, which can help us to estimate the net effect of short-range interaction on microbiota assembly. On the other hand, for most field studies, it is always labor intensive and expensive to collect enough time series for a practical spatial ecosystem. We may sometimes fail in detecting the pattern predicted by a traditional spatial model based on the limited replicates and duration of practical field experiment [26]. In comparison, the Go-board model can provide a simulated snapshot of the community assembly with different interaction patterns. We believe that the combination of the Go-board model and null modeling approach can be used to predict all possible assembly outcomes with complex interactions. Accordingly, the comparison of the null outcomes between 2 communities can help us to estimate the role of space in determining the difference in their structure even with limited number of observations.

Our results suggest that microbial interaction can not only serve as the deterministic factor but also contribute to the stochastic assembly processes, a finding that is contradictory to the traditional niche-based theory [31]. More specifically, strong inhibition on stochastic species can reduce the biodiversity of the local community and unexpectedly lead to the observed dominance of stochastic process. On the other hand, the inhibition on deterministic species can result in enhanced stochasticity during microbiota assembly as expected. We believe that these stochastic effects are attributable to the variation of observed and null βMPDs between 2 communities generated by the short-range interaction on the Go-board. First, consider the interspecific inhibition without the interaction range limitation in a graph. In this case, the inhibition on stochastic species (i.e., *f_sto, u_f_det, v_* and *f_sto, u_f_sto, v_* in eq. 5 approach zero) should result in the observed βMPD close to *d_det, det_* and null βMPDs close to *d_sto, sto_*. By contrast, inhibition on deterministic species (i.e., *f_sto, u_f_det, v_* and *f_det, u_f_det, v_* approach zero) should lead to observed βMPD close to *d_sto, sto_* and null βMPDs close to *d_det, det_*. However, due to short interaction range limitation, the inhibition on stochastic and deterministic species may not lead to the complete extinctions of these species. Consequently, the observed and null βMPDs will approach each other and the observed βMPD is more likely to lie within the 95% RI of the null βMPDs. Accordingly, iCAMP will be inclined to identify the stochastic processes as the dominant process for microbiota assembly.

Based on these findings, we can expect that the facilitation and large phylogenetic distance between deterministic species can cancel out the contribution of short-range inhibition to the stochastic assembly process. Indeed, an experimental study also shows that the variation of interspecific cooperation can diversify the community succession into distinct outcomes [9], which is consistent with our simulation. Overall, the effect of interspecific interaction on microbiota assembly depends not only on the type and phylogenetic relationship of interacting species, but also on the strength and sign of their interaction. Notably, these conclusions were reached based on the assumption that all microbes are static on the Go-board and cannot disperse across the local community. Therefore, the Go-board model may fail to simulate some practical microbiota assemblies (e.g. a fluidic ecosystem) [2]. However, if microbial interaction fails to serve as a stable deterministic factor for microbiota assembly in the ideal environment, it is likely that the niche-based assumption does not hold in the practical environment either. This observation challenges many existing algorithms based on this assumption and it is worth speculating about what the true relative importance of stochasticity is for community assembly.

To date, stochastic process has been regarded as one of the major forces shaping the community structure [31]. The dominance of stochasticity during assembly has been widely observed in microbiota from grassland [27], groundwater [2], ocean sediment [33], wastewater treatment plants (WWTPs) [34, 35], etc. However, our study suggests that such stochasticity may arise from interspecific interaction rather than immigration, random birth, death, and reproduction of microbes themselves. Our study results should help to explain the conflicting reports that activated sludge assembly in WWTPs is driven by deterministic processes in China [36], but by stochastic ones at global scale [34]. We speculate that the microbial interactions in these WWTPs are different due to the different wastewater influent composition [36], thus leading to biased prediction of the assembly process.

Better understanding of the true relative importance of assembly processes is the key for us to design the synthesis strategy of microbiota with desired taxa and functions. For example, the regulation of hydraulic retention time can help us to steer the assembly of stochastic species in biofilm system by manipulating the microbial immigration [37]. Applying specific environmental filtering (e.g. nutrient supplementation or temperature control) should help us to enrich the deterministic species with specific cellular trait [38]. According to our results, the interspecific inhibition between different species could reduce the relative importance of deterministic selection and enhance the stochasticity of microbiota assembly under certain circumstances. Given the ubiquitous microbial competition [39], an urgent need exists for assessing interspecific interaction before the analysis of microbiota assembly processes in the future. Care needs to be taken to ensure the prediction accuracy and precision of null model approaches like iCAMP under different strengths and signs of interspecific interaction. Given the high biodiversity of microbiota in most systems [34], artificial intelligence should be integrated in future analysis to unveil the effects of their interactions on the assembly process. We believe that such investigations will help us to better predict the true assembly process of different microbes and design the microbiota synthesis strategy with more predictable outcome.

## Supplementary Material

ycag043_Supplementary_materials

## Data Availability

All data analysed during this study can be generated by Generate_community_phyloXXX.R in the Supplementary Codes files.
