# Supplementary material for: Potential contribution of microbial interaction to stochasticity of microbiota assembly
Source: ISME Commun. 2026 Feb 27;6(1):ycag043. doi: 10.1093/ismeco/ycag043 (PMC13219740; doi:10.1093/ismeco/ycag043)
Supplement: ycag043_Supplementary_materials [file ycag043_supplementary_materials.zip › Supplementary_materials_ycag043.docx]

Supporting Information for

# Potential Contribution of Microbial Interaction to Stochasticity of Microbiota Assembly

Zhong Yu ^a, b #^, Yupeng Liu ^c, d #^

^a^ School of Environmental Science and Engineering, Sun Yat-sen University, Guangzhou 510275, PR China

^b^ Guangdong Provincial Key Laboratory of Environmental Pollution Control and Remediation Technology (Sun Yat-sen University), Guangzhou 510275, PR China

^c^ Department of Cardiology, Guangdong Provincial People's Hospital (Guangdong Academy of Medical Sciences), Southern Medical University, Guangzhou, China

^d^ Guangdong Cardiovascular Institute, Guangdong Provincial People's Hospital, Guangdong Academy of Medical Sciences, Guangzhou, China

**# Corresponding author**

Zhong Yu, Ph. D.

Email: [yuzh57@mail.sysu.edu.cn](mailto:mengfg@mail.sysu.edu.cn)

Room 401-1, Environmental Building, Sun Yat-sen University, Panyu District, Guangzhou, Guangdong Province, PR China

Yupeng Liu, Ph. D.

Email: [liuyupeng@gdph.org.cn](mailto:liuyupeng@gdph.org.cn)

## Fig S1. The relative importance of homogeneous selection, homogenizing dispersal, and drift in the assembly of simulated microbiota without interaction.

## Fig S2. The relative importance of drift in the assembly of microbiota as functions of the relative abundances of deterministic species and proportion of the inhibited stochastic species in the community.

## Fig. S3. The Shannon indices of simulated microbiota after the inhibition of deterministic species on stochastic ones at *d_det, det_* = 0.5.
